# MRCP surveillance of low-risk branch-duct IPMNs: longitudinal size changes and clinical outcomes in a real-world cohort

**DOI:** 10.1007/s00261-026-05561-7

**Published:** 2026-05-15

**Authors:** Nesrin Gunduz, Huseyin Ertugrul Ozay, Muhammed Furkan Tapan, Fatih Buyuker, Orhan Alimoglu

**Affiliations:** 1 Department of Radiology, Istanbul Medeniyet University, Istanbul, Turkey; 2 Department of General Surgery, Istanbul Medeniyet University, Istanbul, Turkey

**Keywords:** Branch-duct IPMN, Magnetic resonance cholangiopancreatography (MRCP), Pancreatic cystic neoplasms, Growth patterns, Surveillance strategy

## Abstract

**Purpose:**

To evaluate cyst growth patterns, interval radiological changes, and clinical outcomes in patients with low-risk branch-duct intraductal papillary mucinous neoplasms (BD-IPMNs) without baseline worrisome features (WF) or high-risk stigmata (HRS), and to assess whether current surveillance strategies might be individualized in this subgroup.

**Methods:**

In this retrospective single-center study, MRCP examinations performed between 2015 and 2025 were reviewed. Among 601 patients with BD-IPMN, 76 patients without baseline WF/HRS and with at least two MRCP examinations were included. The primary study endpoint was a composite of clinically actionable progression (new WF/HRS, including main pancreatic duct (MPD) dilatation 5–9 mm, or surgical intervention) or study-defined meaningful cyst growth (> 2 mm/year or ≥ 20%/year). Interobserver agreement was assessed using intraclass correlation coefficient (ICC). Descriptive analyses, sensitivity analyses, and exploratory association analyses were performed.

**Results:**

The cohort had a mean age of 65.6 ± 10.7 years, with 58% female patients. The median follow-up duration was 39.5 months (IQR 27.8–72.0). Although follow-up extended longer in a subset of patients, the cohort’s median observation time was approximately 3.3 years., with a median of 3 MRCPs per patient. Median cyst size increased minimally from 10.0 mm to 11.0 mm, with a median annual growth of 0.11 mm/year. The composite endpoint occurred in 9 patients (11.8%), comprising 5 clinically actionable events (3 BD-IPMN-related WF and 2 MPD dilatation cases classified as WF) and 4 cases of meaningful cyst growth without other WF/HRS. No patient developed HRS or underwent surgical intervention. No patient developed remote PDAC during follow-up. Interobserver agreement was excellent (ICC ≥ 0.93).

**Conclusion:**

Low-risk BD-IPMNs demonstrated minimal growth and infrequent clinically actionable progression during MRCP surveillance. These findings are consistent with an indolent course, particularly in patients with small cysts, but should be interpreted in the context of limited event counts, non-standardized contrast use, selection bias, and a median follow-up of approximately 3.3 years. Larger multicenter studies with standardized imaging and longer follow-up are needed before specific surveillance de-escalation strategies can be recommended.

**Supplementary Information:**

The online version contains supplementary material available at https://doi.org/10.1007/s00261-026-05561-7.

## Introduction

Intraductal papillary mucinous neoplasms (IPMNs) of the pancreas are well-established precursor lesions of pancreatic ductal adenocarcinoma and are increasingly detected owing to the widespread use of cross-sectional imaging and magnetic resonance cholangiopancreatography (MRCP) [1, 2]. Among the recognized subtypes, branch-duct IPMNs (BD-IPMNs) represent the most frequently encountered form in clinical practice. Although BD-IPMNs carry a substantially lower risk of malignant transformation compared with main-duct or mixed-type IPMNs, their long-term management and optimal surveillance strategy remain subjects of ongoing debate [3, 4].

Current international consensus guidelines, most notably the revised Fukuoka criteria, recommend structured surveillance intervals aimed at early detection of worrisome features (WF) or high-risk stigmata (HRS) that may prompt surgical intervention [1]. While these recommendations prioritize oncologic safety, they are derived from heterogeneous cohorts and intentionally adopt a conservative approach. Consequently, concerns have emerged regarding potential over-surveillance, particularly among patients with BD-IPMNs lacking baseline WF or HRS, in whom the absolute risk of progression appears to be low [5, 6].

Recent multicenter cohorts [7, 8] and evolving guidelines [9] have proposed that surveillance intervals may be individualized based on baseline cyst size and interval stability, and that surveillance discontinuation might be considered in selected older patients after prolonged stability. However, the evidence remains heterogeneous, and long-term management is complicated by the potential risk of concomitant (remote) pancreatic ductal adenocarcinoma developing elsewhere in the pancreas, independent of the index cyst. .

Frequent long-term imaging surveillance may impose a substantial burden on healthcare systems, increase costs, and contribute to patient anxiety without a clearly demonstrated clinical benefit. Therefore, robust real-world data examining cyst growth patterns, radiological evolution, and clinical outcomes in low-risk BD-IPMN populations are needed to inform more individualized surveillance strategies.

The aim of this study was to evaluate cyst growth patterns, interval radiological changes, and clinical outcomes in patients with BD-IPMNs without baseline WF or HRS who underwent serial MRCP surveillance over a 10-year period. In particular, we sought to assess whether the surveillance intensity recommended by current guidelines may be safely reduced in this low-risk subgroup.

## Materials and methods

This retrospective single-center study was approved by the local institutional review board, and the requirement for informed consent was waived due to the retrospective nature of the analysis. The medical records of patients diagnosed with branch-duct intraductal papillary mucinous neoplasms (BD-IPMNs) between January 2015 and December 2025 at a tertiary referral center were reviewed.

**Fig. 1:**
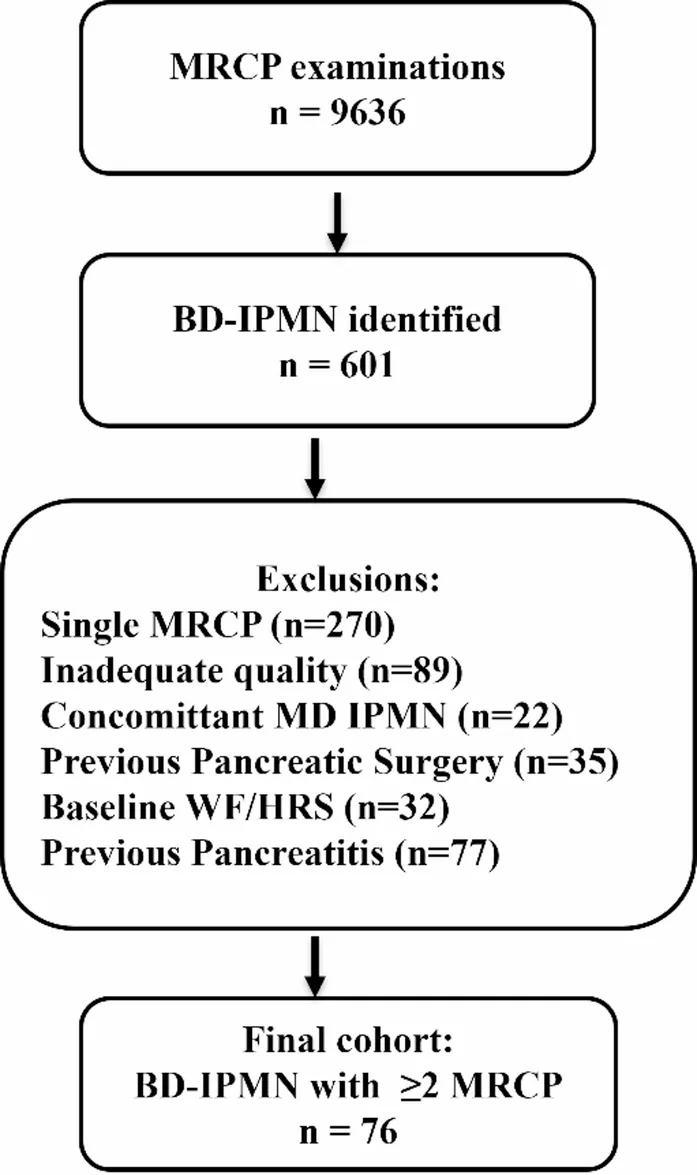
Flow diagram illustrating patient selection and exclusion criteria for the study cohort

Over a 10-year period, 601 patients were identified with imaging findings consistent with branch-duct IPMN. Of these, 270 patients were excluded due to having only a single MRCP examination, 89 due to inadequate MRCP image quality for confident diagnosis, 22 due to the presence of concomitant main-duct or mixed-type IPMN, 35 due to a history of pancreatic surgery, 32 due to the presence of worrisome features or high-risk stigmata at baseline, and 77 due to a history of pancreatitis. After applying these exclusion criteria sequentially, a total of 76 patients with branch-duct IPMN who had at least two MRCP examinations and no baseline worrisome features or high-risk stigmata were included in the final analysis (Fig. 1).

Inclusion criteria were: (1) BD-IPMN diagnosis confirmed by magnetic resonance cholangiopancreatography (MRCP);

(2) absence of worrisome features (WF) or high-risk stigmata (HRS) at baseline according to the 2017 revised Fukuoka guidelines; and.

(3) availability of at least two MRCP examinations during follow-up.

The 2017 revised Fukuoka criteria were used as the primary methodological framework because they were the prevailing standard during the study period and they ensured consistency in baseline classification and follow-up assessment.

### Imaging protocol and image analysis

**Fig. 2:**
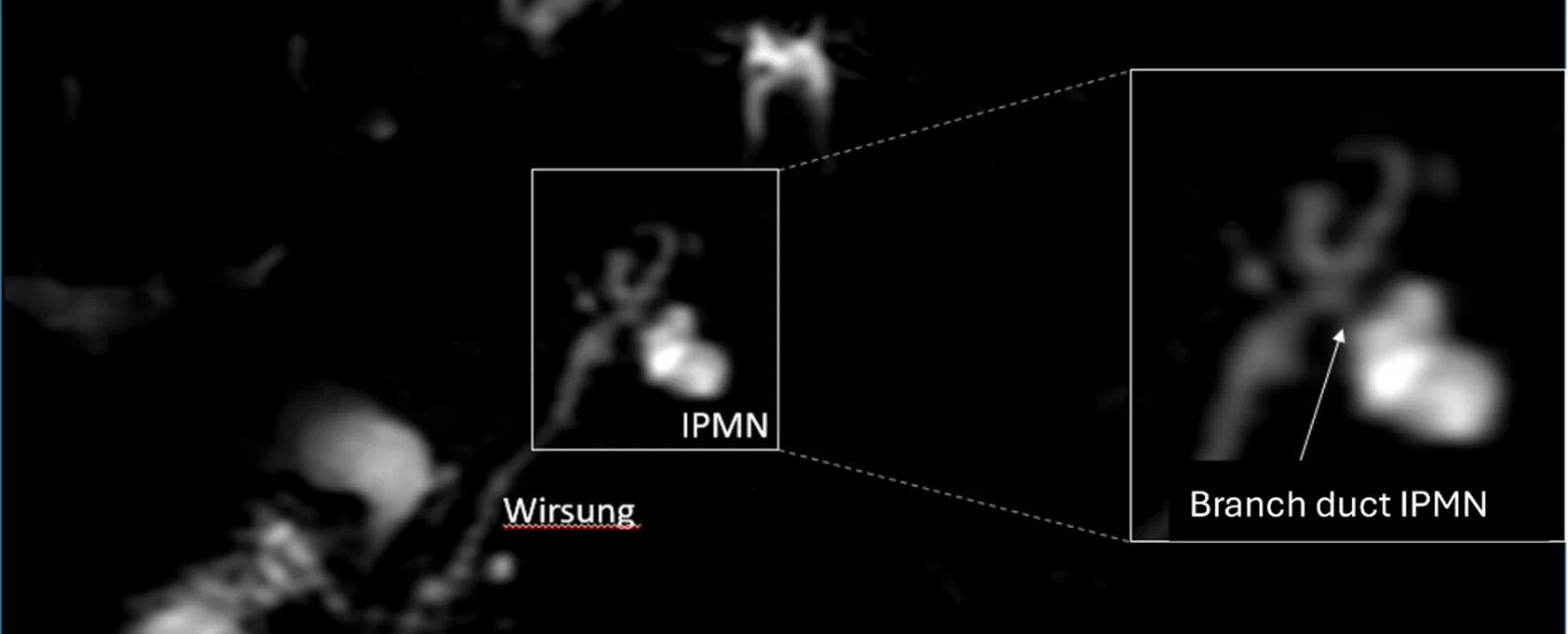
The pancreatic cyst is shown to communicate with the main pancreatic duct, consistent with a branch-duct IPMN

MRCP examinations were performed using 1.5-T MRI systems following standard institutional pancreatic imaging protocols, including heavily T2-weighted MRCP sequences and contrast-enhanced abdominal MRI when clinically indicated. All MRCP images were reviewed separately by two radiologists with more than 10 and 5 years of experience in abdominal imaging, respectively, blinded to the clinical data and previous radiological reports. Thereafter, disagreements were resolved by consensus. Branch-duct IPMN was defined as a unilocular or multilocular (grape-like) pancreatic cysts, typically < 3 cm, that communicate with a non-dilated main pancreatic duct (< 5 mm) on MRCP, with pseudocyst considered in the differential diagnosis in patients with a prior history of pancreatitis. These lesions are often located in the uncinate process, though they can appear throughout the gland. They do not contain a thick, fibrous/calcified capsula. A demonstrable connection (short neck or channel) to main pancreatic duct (MPD) is the key diagnostic feature that differentiates it from other cystic lesions of the pancreas. (Fig. 2).

Contrast-enhanced abdominal MRI was performed when clinically indicated rather than according to a uniform study protocol. Accordingly, contrast use was non-standardized across examinations and may have influenced the sensitivity for detecting enhancing mural nodules or subtle wall enhancement. The proportion of patients/examinations performed with gadolinium at baseline and follow-up is reported in Supplementary Table 1.

To further characterize MRI protocol heterogeneity, we retrospectively recorded patient-level gadolinium exposure, including whether a contrast-enhanced MRI was performed, the number of contrast-enhanced examinations, and the approximate interval between repeated contrast-enhanced studies. In our institutional registry, a single contrast-enhanced examination indicated baseline-only gadolinium exposure, whereas more than one contrast-enhanced examination indicated repeated gadolinium-enhanced follow-up during surveillance.

The number of MRCP examinations performed was recorded.

Cyst location was classified as uncinate process, head, corpus, or tail of the pancreas. The diameter of the largest cystic lesion was measured in millimeters on axial or coronal images at baseline and at the final follow-up MRCP. When multiple cysts were present, the dominant (largest) cyst was used for growth analysis.

**Fig. 3:**
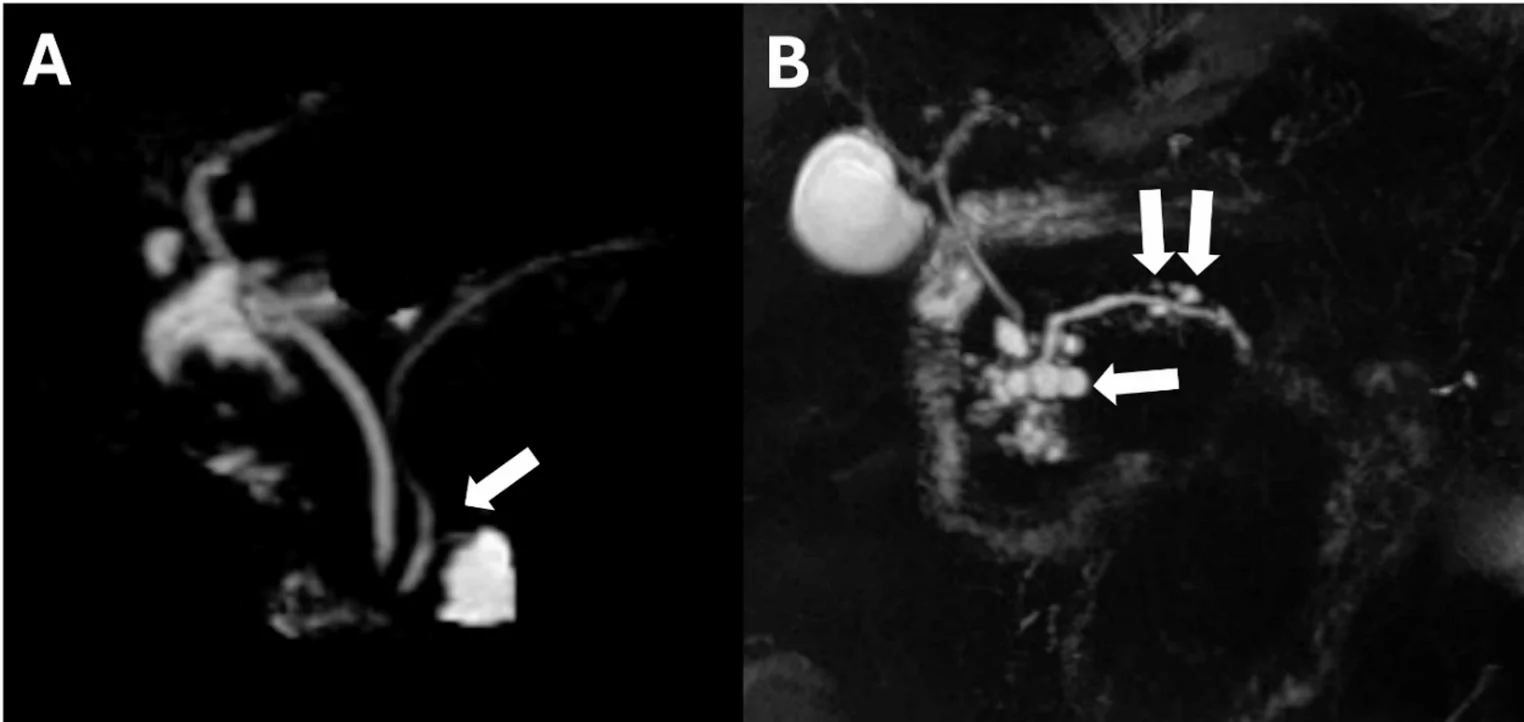
**A** MR cholangiopancreatography (MRCP) image demonstrating a branch-duct intraductal papillary mucinous neoplasm (BD-IPMN) (arrow) located in the uncinate process, the most common site of involvement for BD-IPMNs. **B** MRCP image showing multifocal IPMNs (arrows) scattered throughout the pancreas, predominantly located adjacent to the main pancreatic duct

Multifocality was defined as the presence of cysts in more than one pancreatic segment, while multiloculation was defined as the presence of internal septations within a cyst (Fig. 3). Follow-up MRCP images were re-evaluated to identify interval radiological changes in BD-IPMNs, including newly developed multifocality, main pancreatic duct dilatation.

**Fig. 4:**
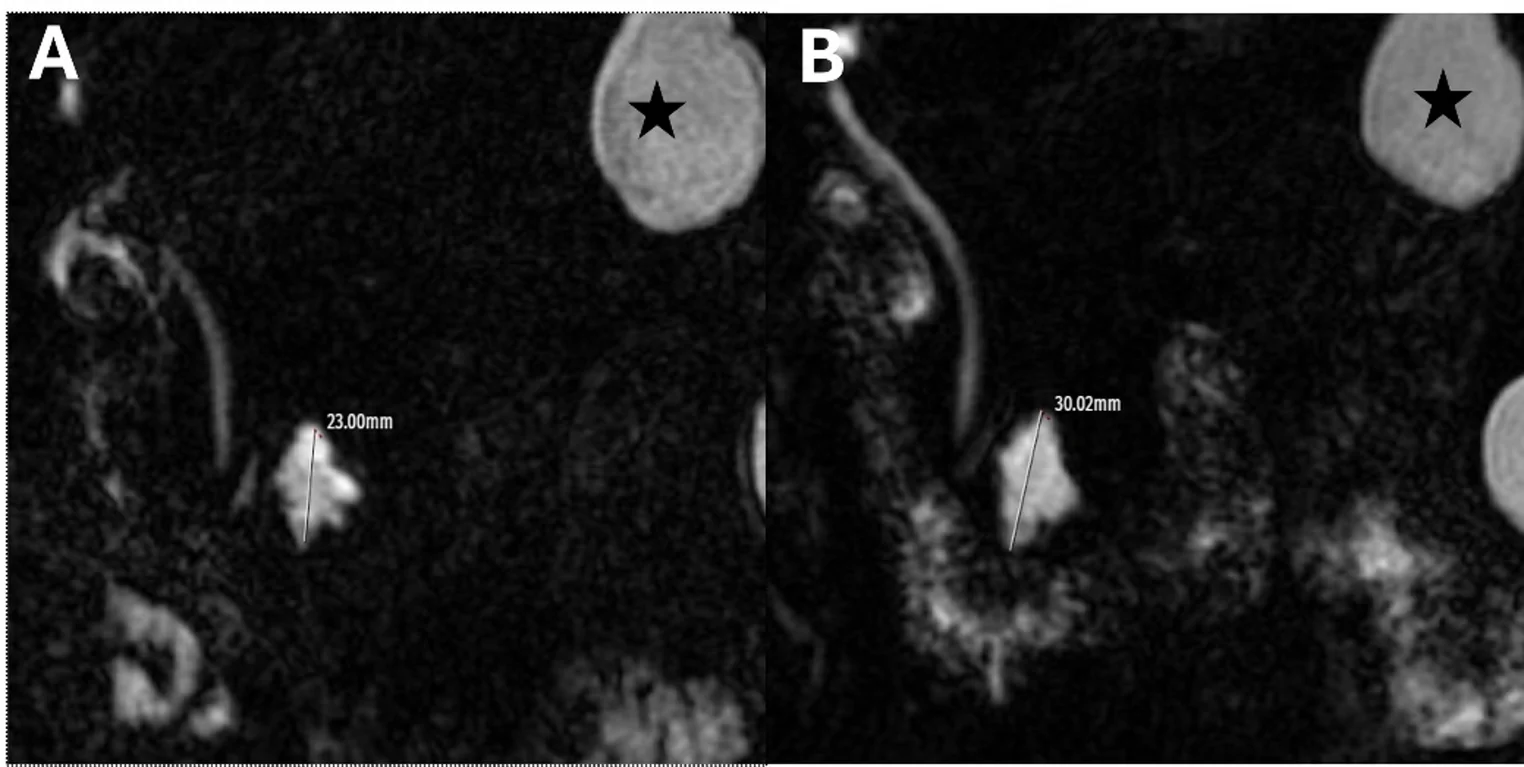
**A** Baseline MRCP demonstrating the largest diameter of BD-IPMN. Asterisk indicates a large renal cyst. **B** Follow up MRCP after 21 months demonstrating size increase to 30 mm (largest diameter) which was considered as WF. Asterisk indicates a large renal cyst

Cyst measurements were performed using electronic calipers on the institutional PACS. For each examination, the maximum diameter of the dominant cyst was recorded in the imaging plane and sequence that best depicted the entire lesion; when feasible, follow-up measurements were repeated on the same plane/sequence to improve consistency (Fig. 4). Given known measurement variability in pancreatic cyst sizing, we interpreted minimal changes cautiously and performed sensitivity analyses using an annualized ≥ 2 mm or ≥ 20% increase threshold to define meaningful progression [10–11]. Meaningful cyst growth was a study-defined radiological threshold intended to reduce the influence of trivial measurement fluctuation and standardize growth assessment across heterogeneous follow-up durations. This threshold should not be interpreted as synonymous with guideline-defined worrisome features.

Interobserver agreement was assessed using the intraclass correlation coefficient (ICC) based on a two-way random-effects model with absolute agreement.

Imaging review systematically assessed mural nodules (including sub-threshold nodules), cyst wall thickening/enhancement, subtle MPD diameter changes, and lymphadenopathy at baseline and follow-up.

Definitions (2017 revised Fukuoka framework) of High-risk Stigmata (HRS) were: (i) obstructive jaundice in a patient with a cystic lesion in the pancreatic head, (ii) enhancing mural nodule ≥ 5 mm, and (iii) main pancreatic duct (MPD) diameter ≥ 10 mm.

Definitions (2017 revised Fukuoka framework) of Worrisome Features (WF) were: (i) pancreatitis, (ii) cyst diameter ≥ 3 cm, (iii) enhancing mural nodule < 5 mm, (iv) thickened/enhancing cyst walls, (v) MPD diameter 5–9 mm, (vi) abrupt change in MPD caliber with distal pancreatic atrophy, (vii) lymphadenopathy, (viii) elevated serum CA19-9, and (ix) cyst growth rate > 5 mm/2 years.

Any interval development of WF or HRS during surveillance was recorded. Moreover de novo development of cysts at remote sites (either main or branch duct) of pancreas were also recorded. Surgical referral and intervention, if any, were documented from clinical records.

Follow-up duration was defined as the interval between the first qualifying (‘baseline’) MRCP and the last available MRCP. Surveillance intensity was operationalized as the total number of MRCP examinations during follow-up (and, when applicable, the average interval between examinations.

The primary endpoint was defined as a composite of [1] clinically actionable interval progression, including development of WF or HRS according to the 2017 revised Fukuoka framework (with MPD dilatation 5–9 mm classified as a WF) or a need for surgical intervention, or [2] study-defined meaningful cyst growth in the absence of other WF/HRS. Meaningful cyst growth was defined as annualized cyst growth > 2 mm/year or a relative increase ≥ 20% per year. This study-specific radiological threshold was selected to reduce the influence of trivial interval fluctuation and to standardize growth assessment across variable follow-up durations; it should not be interpreted as synonymous with guideline-defined WF.

### Statistical analysis

Descriptive statistics were presented as mean ± SD, median with interquartile range (IQR), or frequencies (%). Absolute growth was defined as the change in maximum cyst size between baseline and last MRCP. Annualized growth was calculated by dividing absolute growth by follow-up duration (months) and multiplying by 12. Surveillance density was calculated by dividing the number of MRCP examinations by follow-up duration (months) and multiplying by 12. Mann–Whitney U was used for independent group comparisons of continuous variables without normal distribution. Spearman correlation coefficients were used to assess associations Interobserver agreement was assessed using a two-way random-effects ICC with absolute agreement. Because of low event rate, risk of overfitting a penalized logistic regression model using Lasso regularization was used. The prespecified predictors of primary end point were selected as baseline cyst size and presence of multifocality. Model performance was assessed using area under the receiver operating characteristic curve (AUC). Odds ratios (ORs) were derived from model coefficients A p value less than 0.05 was accepted as significant for all analyses. A pairwise deletion approach was used for missing data because missing data was very small (less than 5%). Analyses were conducted using SPSS v.26.0 (IBM Corp, Armonk, NY).

## Results

A total of 221 MRCP examinations were performed in the 76 patients during the 10-year study period.

A total of 76 patients with BD-IPMN fulfilled the inclusion criteria. The mean age was 65.6 ± 10.7 years, and 58% were female. The median follow-up duration was 39.5 months (IQR 27.8–72.0), with a median of 3 MRCP scans per patient (IQR 2–3). The baseline median cyst size was 10.0 mm (IQR 6.0–15.0), which increased minimally to 11.0 mm (IQR 7.0–16.0) at the last follow-up. The median absolute growth was 0.0 mm (IQR 0.0–3.25), corresponding to a median annualized growth of 0.11 mm/year (IQR 0.00–0.70) (Table 1). Multifocality was observed in 39% of patients, multiloculation in 15%, but no significant correlation with growth rate was identified.

**Table 1:** Baseline demographic and imaging characteristics of the study cohort (*n* = 76)

Variable	Value
Age, mean ± SD (years)	65.6 ± 10.7
Female sex, n (%)	44 (58%)
Baseline cyst size, median (IQR), mm	10.0 (6.0–15.0)
Follow-up duration, median (IQR), months	39.5 (27.8–72.0)
Number of MRCP examinations, median (IQR)	3 (2–3)
Multifocality, n (%)	30 (39%)
Multiloculation, n (%)	11 (15%)

SD, standard deviation; IQR, interquartile range; MRCP, magnetic resonance cholangiopancreatography

Interobserver agreement for cyst size measurements was excellent, with an intraclass correlation coefficient (ICC) of 0.93 for the baseline cyst size and 0.94 for last MRI follow up measurements.The surveillance intensity was calculated as 1.2 ± 1.1 MRIs per year.

The primary outcome was observed in 9 (11.8%) patients [5 (6.6%) clinically actionable events and 4 (5.2%) pure radiological progression]. Of these 3 patients developed BD-IPMN-related worrisome features (2 lesions reaching ≥ 30 mm and 1 lesion developing thickened/enhancing cyst walls), and 2 developed MPD dilatation of 5–9 mm, which was classified as a worrisome feature. The remaining 4 patients met the prespecified criteria for meaningful cyst growth without other WF/HRS (Table 2). No patient developed HRS or underwent surgical intervention. All 3 patients who developed BD-IPMN-related WF underwent endoscopic ultrasound, and cytology was benign in all cases.

**Table 2:** Cases meeting study endpoints

Case	Baseline BD-IPMN size (mm)	Time to Outcome (months)	Outcome type	Management
1	23	21	WF (BD-IPMN ≥ 30 mm)	Referred for EUS
2	28	34	WF (BD-IPMN ≥ 30 mm)	Referred for EUS
3	16	33	WF (thickened/enhancing cyst walls of BD-IPMN)	Referred for EUS
4	7.5	72	BD-IPMN meaningful cyst growth	Continued surveillance
5	13.8	54	BD-IPMN meaningful cyst growth	Continued surveillance
6	19	6	BD-IPMN meaningful cyst growth	Continued surveillance
7	29.5	38	BD-IPMN meaningful cyst growth	Continued surveillance
8	21.4	77	WF (MPD dilation)	Continued surveillance (advanced age and comorbidities)
9	22.7	39	WF (MPD dilation)	Continued surveillance (advanced age and comorbidities)

When stratified according to baseline cyst size; 1 (3.2%) of 31 cysts < 10 mm in size developed primary outcome, 3 (9%) of 33 cysts ≥ 10 mm to < 20 mm in size developed primary outcome, 5 (41.7%) of 12 cysts ≥ 20 mm to < 30 mm developed primary outcome. Main pancreatic duct dilatation consistent with concomitant main-duct IPMN (duct diameter 5–10 mm) developed in 2 (2.63%) patients during follow up. The baseline IPMN cyst sizes were 21.4 mm and 22.7 mm, respectively. Given their advanced age (74 and 75 years) and comorbidities, they were managed with continued surveillance with MRCP rather than endoscopic ultrasound or surgical intervention. Those with prespecified meaningful BD-IPMN growth were not considered as candidates for any invasive strategy and managed only by follow-up MRIs as per guidelines.

For sensitivity analysis purpose, when cases with a relatively low period of follow-up (i.e. less than 24 months, 18 patients) are not taken into account, 8 (13.6%) of 59 patients developed primary outcome.

No correlations were observed between annualized absolute growth rate and age (*r* = 0.161, *p* = 0.16) as well as surveillance density (*r* = 0.15, *p* = 0.18).

No patient developed pancreatic ductal adenocarcinoma remote from the index cyst during the observation period. During follow-up, 8 patients (10.5%) developed de novo cysts at remote sites. All de novo lesions were branch-duct type. Their size at first detection was median 6.3 mm (range between 4.7 and 8.7 mm). None of the de novo cysts developed WF during the study period.

In the penalized regression model including baseline cyst size and multifocality, model discrimination was moderate (AUC: 0.75). Baseline cyst size was the only significant predictor of the composite endpoint (OR: 2.30, 95% CI: 1.25–4.10, *p* = 0.008), whereas multifocality showed no significant association.

Overall 70 of 76 patients (92.1%) underwent at least one contrast-enhanced MRI during surveillance, corresponding to baseline contrast-enhanced imaging in our registry. Seven patients (9.2%) had only a single baseline contrast-enhanced examination, whereas 63 patients (82.9%) underwent more than one contrast-enhanced MRI during follow-up. Among those with repeated contrast-enhanced MRI, the median interval between enhanced examinations was 12 months (IQR, 6–12 months). Seven patients (9.2%) underwent contrast-enhanced MRI partly because of another disease or malignancy-related indication.

## Discussion

This retrospective single-center study provides further insight into the natural history of BD-IPMNs without baseline WF or HRS. The principal finding is current study’s low risk lesions follow anindolent clinical course, characterized by minimal cyst growth, a relatively low incidence of newly developed WF, complete absence of HRS, and no requirement for surgical intervention during up to ten years of follow-up.

According to the revised 2017 Fukuoka guidelines, surveillance intervals are primarily stratified by cyst size, ranging from 18 to 24 months for cysts smaller than 2 cm to as frequently as every 3–6 months for cysts larger than 3 cm [1]. Although such recommendations are designed to minimize the risk of missed malignant transformation, our findings suggest that this degree of surveillance intensity may not be necessary for BD-IPMNs lacking baseline risk features. In our cohort, cysts < 10 mm showed a very low event rate (1/31, 3.2%), and no patient in this subgroup developed WF, HRS, or required surgery during follow-up. Moreover penalized regression analysis suggested that only the baseline cyst size remains the primary determinant of risk. These observations are directionally consistent with recent literature suggesting that selected patients with small, stable BD-IPMNs may be candidates for less intensive follow-up. However, our data should be viewed as hypothesis-generating rather than practice-changing, because the overall median follow-up was 39.5 months and only a subset of patients had substantially longer observation. Accordingly, our findings support consideration of individualized surveillance in carefully selected patients with small, stable cysts, rather than routine surveillance cessation based on our data alone. By contrast, baseline cysts measuring 10–19 mm showed an intermediate event rate (3/33, 9.1%), while cysts measuring 20–29 mm showed a substantially higher event rate (5/12, 41.7%), supporting continued size-stratified surveillance in larger lesions.

Our primary methodological framework was Fukuoka 2017 for consistency across the study period. However, case level contextualization of our 9 patients developing study endpoints using Kyoto 2024 criteria is also available in Supplementary Tables 2 and 3.

Contrast-enhanced abdominal MRI was performed according to clinical judgment rather than a fixed surveillance schedule, and repeated contrast-enhanced imaging was partly attributable to other oncologic indications in a subset of patients. Because the Fukuoka 2017 and Kyoto 2024 frameworks rely in part on enhancement-based features, such as enhancing mural nodules and thickened/enhancing cyst walls, this heterogeneity may have reduced sensitivity for enhancement-defined worrisome feature ascertainment and may therefore have biased the observed worrisome feature rate downward. This concern is particularly relevant for subtle mural nodules or early wall enhancement, although many surveillance outcomes in our cohort, including cyst size progression and main pancreatic duct dilatation, remain assessable on non-contrast MRCP.

More recent international evidence-based guideline updates have further refined risk stratification by incorporating endoscopic ultrasound (EUS) findings and cytological analysis into the assessment of HRS and WF. Importantly, these guidelines introduce the option of discontinuing surveillance after five years in small, stable BD-IPMNs, while acknowledging the persistent possibility of concomitant pancreatic ductal adenocarcinoma [9]. Our results strongly align with this evolving perspective, as none of the patients in our low-risk cohort progressed to HRS or required surgical intervention despite extended follow-up, thereby.

providing support for a more individualized and potentially de-escalated surveillance approach.

Overall cyst growth in our cohort was negligible, with a median annualized growth rate of only 0.11 mm/year, which is well below the magnitude of measurement variability reported for pancreatic cyst sizing on cross-sectional imaging. Prior work has demonstrated median size differences on the order of several millimeters between imaging modalities (e.g., CT vs. MRI) even when studies are performed within short intervals. Therefore, the minimal changes observed here should be interpreted primarily as radiological stability rather than definitive biological growth [10]. Moreover four “meaningful growth only” cases may represent either early biological change or radiological variability, and that these events were included as study-defined radiologic progression rather than as equivalent clinical progression.

Only three patients developed BD-IPMN WF all of which with benign EUS and cytology findings, and no patient developed HRS. These observations are consistent with prior long-term studies reporting annual malignant transformation rates below 0.5% in BD-IPMNs without WF or HRS, reinforcing the concept that this subgroup represents a particularly low-risk population. A recent meta-analysis further confirmed that stable low-risk BD-IPMNs exhibit a very low incidence of advanced neoplasia during extended surveillance, particularly after five years of size stability [12]. In addition, large cohort data demonstrate that baseline cyst size is a key predictor of WF and HRS development, supporting size-based stratification of surveillance intervals in low-risk BD-IPMNs [13]. Similarly, Lee et al. showed that BD-IPMNs without interval change during the first five years have an exceedingly low subsequent risk of malignant transformation, highlighting radiological stability as a powerful predictor of long-term indolent behavior [8].

Risk-adapted de-escalation of surveillance for stable BD-IPMN must be balanced against the concept of dual carcinogenesis, whereby pancreatic ductal adenocarcinoma may arise elsewhere in the gland independent of the index cyst. In our cohort, no patient developed remote PDAC, but de novo pancreatic cysts developed in 8 patients (10.5%), supporting the concept that surveillance should remain attentive to the whole pancreas rather than only to the index lesion. This is particularly relevant in the setting of multifocal disease, which has been linked in prior studies to concomitant pancreatic neoplasia. The Kyoto 2024 guidelines highlight this concern and summarize evidence suggesting that the cumulative risk of concomitant PDAC may increase over time, with reported annual incidence on the order of 0.4–1.0% in some series, although regional differences exist. This consideration may justify continued whole-pancreas surveillance in selected patients even in the absence of cyst progression [9].

The absence of a significant correlation between surveillance density and annualized growth does not exclude surveillance bias. Imaging frequency was partly determined by clinical judgment and perceived risk; therefore, this finding only indicates that MRCPs per year were not linearly associated with measured growth in this cohort. Minor interval changes should still be interpreted cautiously because small apparent growth may reflect radiological variability rather than true biological progression.

Although multifocality and multiloculation were not significantly associated with cyst growth in our cohort, prior studies have suggested that multifocal disease may reflect a field defect within the pancreas and could be associated with an increased risk of progression or concomitant neoplasia [14]. However, their independent prognostic value remains inconsistent across studies and warrants further investigation .

Although our study did not perform a formal cost-effectiveness analysis, the observation that 221 MRCP examinations were performed in this low-risk cohort without any surgical intervention raises the hypothesis that resource utilization might be optimized through more individualized surveillance intervals in carefully selected patients. This observation should be regarded as hypothesis-generating and not as a direct economic conclusion from our data. Supporting this perspective, a UK-based cohort study demonstrated that patients with stable BD-IPMNs for at least five years had pancreatic cancer risks comparable to the general population, and surveillance discontinuation in selected individuals was associated with substantial annual cost savings [15]. Likewise, a large international multicenter study reported that patients with presumed BD-IPMNs without WF or HRS who remained stable for at least five years had standardized incidence ratios comparable to age-matched controls, with surveillance discontinuation considered justifiable in older patients and those with small cysts [16].

Collectively, these findings support a shift toward a more individualized, risk-adapted surveillance strategy for BD-IPMNs. Rather than strict adherence to uniform guideline-based schedules, follow-up intervals should incorporate baseline cyst size, interval stability, patient age, and comorbidities. In line with this concept, the PANORAMA calculator demonstrated that age and cyst size significantly influence the timing of IPMN evolution and suggested that routine semestral or annual follow-up may be unnecessarily intensive after an initial six-month reassessment in selected low-risk patients [17].

Limitations: Several limitations merit emphasis. First, this was a retrospective single-center study, which limits generalizability. Second, only 9 patients met the primary endpoint; therefore, any regression analysis should be regarded as exploratory because the low number of events increases the risk of overfitting and unstable effect estimates. Third, inclusion required at least two MRCP examinations, introducing potential survivorship/selection bias by preferentially retaining patients who completed structured surveillance. Fourth, EUS was performed selectively rather than uniformly, and cyst fluid cytology has limited sensitivity; therefore, negative cytology does not exclude low-grade or intermediate-grade dysplasia. Because no patient underwent surgery, histopathological confirmation was unavailable in this cohort. Finally, the median follow-up was 39.5 months, so our findings primarily reflect short- to intermediate-term surveillance, although some patients were observed longer. An additional major limitation is the lack of a standardized gadolinium protocol. Accordingly, the low rate of worrisome feature development in this cohort should be interpreted with caution, particularly for enhancement-defined features, which may have been under-ascertained in the absence of a standardized contrast-enhanced MRI protocol.

## Conclusion

In this retrospective single-center cohort of patients undergoing structured MRCP surveillance for low-risk BD-IPMN, clinically actionable progression was uncommon, no patient developed HRS or required surgery, and lesions < 10 mm showed particularly low event rates. However, larger baseline cysts—especially those measuring 20–29 mm—were not risk-free. These findings are consistent with an indolent course in carefully selected patients, but should be interpreted as hypothesis-generating given the limited number of events, non-standardized contrast use, selection bias, and the cohort’s median follow-up duration. Larger multicenter studies with standardized imaging protocols and longer follow-up are required before recommending specific surveillance de-escalation or discontinuation strategies.

## Supplementary Information

Below is the link to the electronic supplementary material.

Supplementary Material 1

Supplementary Material 2

Supplementary Material 3

## Data Availability

The datasets generated and/or analyzed during the current study are not publicly available due to patient confidentiality but are available from the corresponding author on reasonable request.
