# Supplementary material for: MRCP surveillance of low-risk branch-duct IPMNs: longitudinal size changes and clinical outcomes in a real-world cohort
Source: Abdom Radiol (NY). 2026 May 15;51(11):5902–10. doi: 10.1007/s00261-026-05561-7 (PMC13672555; doi:10.1007/s00261-026-05561-7)
Supplement: Supplementary file 1 [file 261_2026_5561_MOESM1_ESM.docx]

**Supplementary Table S3. Case-level interpretation of the 9 study endpoint cases under the Kyoto 2024 framework**

This supplementary table presents a case-level reclassification of the nine patients who met the primary composite endpoint in our study, interpreted according to the 2024 Kyoto guidelines. This comparison aims to contextualize our findings within updated risk stratification criteria and to distinguish between radiological progression and clinically actionable events.

| **Case** | **Baseline cyst size (mm)** | **Outcome (study-defined)** | **Kyoto 2024 classification** | **Clinically actionable event** | **Interpretation** |
| --- | --- | --- | --- | --- | --- |
| 1 | 23 | WF (cyst ≥30 mm) | WF | Yes | Meets WF criteria in both frameworks; clinically relevant progression |
| 2 | 28 | WF (cyst ≥30 mm) | WF | Yes | Consistent WF under Kyoto; size-driven progression |
| 3 | 16 | WF (wall thickening/enhancement) | WF | Yes | Concordant WF classification; clinically actionable |
| 4 | 7.5 | Growth-only | Possible WF (≥2.5 mm/year depending on annualized rate) | No | Likely reflects measurement variability rather than clinically meaningful progression |
| 5 | 13.8 | Growth-only | Possible WF | No | Borderline case; classification depends on growth calculation method |
| 6 | 19 | Growth-only | Possible WF | No | May meet Kyoto growth threshold; no additional WF |
| 7 | 29.5 | Growth-only | Possible WF | No | Growth near upper size range; still no WF/HRS |
| 8 | 21.4 | MPD dilation (5–9 mm) | WF | Yes | Classified as WF under both frameworks |
| 9 | 22.7 | MPD dilation (5–9 mm) | WF | Yes | Concordant WF classification |

**Abbreviations:** WF, worrisome features; HRS, high-risk stigmata; MPD, main pancreatic duct.

When reclassified according to the Kyoto 2024 framework, the majority of clinically meaningful events (n=5) remain consistent with worrisome features, including cyst size progression, mural changes, and main pancreatic duct dilatation. However, the four cases categorized as “growth-only” in our study represent a distinct subgroup in which classification depends on the application of annualized growth thresholds introduced in the Kyoto guidelines. Importantly, none of these cases were associated with additional worrisome features, high-risk stigmata, or surgical intervention, suggesting that they may reflect measurement variability rather than true biological progression. This distinction highlights the importance of interpreting growth thresholds cautiously and supports separating radiological progression from clinically actionable events in surveillance studies.
