# Supplementary material for: MRCP surveillance of low-risk branch-duct IPMNs: longitudinal size changes and clinical outcomes in a real-world cohort
Source: Abdom Radiol (NY). 2026 May 15;51(11):5902–10. doi: 10.1007/s00261-026-05561-7 (PMC13672555; doi:10.1007/s00261-026-05561-7)
Supplement: Supplementary file 2 [file 261_2026_5561_MOESM2_ESM.docx]

**Supplementary Table S1. Patient-level gadolinium exposure during MRI surveillance**

| **Variable** | **Value** |
| --- | --- |
| Patients with at least one contrast-enhanced MRI during surveillance | 70/76 (92.1%) |
| Patients with no contrast-enhanced MRI during surveillance | 6/76 (7.9%) |
| Patients with only one contrast-enhanced MRI (baseline only, by registry definition) | 7/76 (9.2%) |
| Patients with more than one contrast-enhanced MRI during surveillance | 63/76 (82.9%) |
| Interval between repeat contrast-enhanced MRI examinations among patients with >1 contrast-enhanced MRI, median (IQR), months | 12 (6–12) |
| Repeat contrast interval of 12 months | 31/63 (49.2%) |
| Repeat contrast interval of 6 months | 14/63 (22.2%) |
| MRI performed partly because of another disease/malignancy indication | 7/76 (9.2%) |
| Other disease/malignancy indications | Ovarian cancer surveillance; breast cancer; endometrial cancer; cholangiocarcinoma; gastric cancer; hepatocellular carcinoma; rectal cancer (one patient each) |

This supplementary table summarizes patient-level gadolinium exposure patterns during MRI surveillance in the study cohort. It provides detailed information on contrast administration frequency, interval between contrast-enhanced examinations, and the presence of additional clinical indications that may have influenced MRI utilization.

Gadolinium-enhanced MRI was performed in the majority of patients, with 92.1% receiving at least one contrast-enhanced examination. Most patients (82.9%) underwent multiple contrast-enhanced MRIs, with a median interval of 12 months, reflecting a relatively structured surveillance approach. However, a small subset of patients (7.9%) did not receive contrast-enhanced imaging, and 9.2% had only a baseline contrast-enhanced MRI, indicating variability in imaging protocols. Additionally, MRI examinations were performed in a minority of patients for indications unrelated to IPMN surveillance, which may have influenced imaging frequency. This heterogeneity in contrast administration should be considered when interpreting the detection rate of enhancement-dependent features such as mural nodules.

**Abbreviations:** MRI, magnetic resonance imaging; IQR, interquartile range.
