# Supplementary material for: MRCP surveillance of low-risk branch-duct IPMNs: longitudinal size changes and clinical outcomes in a real-world cohort
Source: Abdom Radiol (NY). 2026 May 15;51(11):5902–10. doi: 10.1007/s00261-026-05561-7 (PMC13672555; doi:10.1007/s00261-026-05561-7)
Supplement: Supplementary file 3 [file 261_2026_5561_MOESM3_ESM.docx]

| **Domain** | **Fukuoka 2017** | **Kyoto 2024** | **Implications for the present cohort** |
| --- | --- | --- | --- |
| **High-risk stigmata (HRS)** | Obstructive jaundice; enhancing mural nodule ≥5 mm; main pancreatic duct (MPD) ≥10 mm | Same core criteria + suspicious or positive cytology if available | No cytology-based HRS were identified in our cohort; the two cases with MPD dilatation remained within the WF category and did not meet HRS criteria |
| **Worrisome features (WF)** | Pancreatitis; cyst ≥30 mm; mural nodule <5 mm; thickened/enhancing wall; MPD 5–9 mm; abrupt caliber change with distal atrophy; lymphadenopathy; elevated CA19-9; growth >5 mm/2 years | Largely similar criteria + new-onset or worsening diabetes mellitus and growth ≥2.5 mm/year | The four “growth-only” cases in our cohort may meet Kyoto WF criteria depending on annualized growth calculations, or may remain classified as study-defined radiological progression |
| **Surveillance discontinuation** | No clearly defined discontinuation pathway | Discontinuation may be considered after 5 years in stable cysts <20 mm, depending on patient condition and life expectancy | Our findings provide directional support for this concept; however, given the median follow-up duration, our data are not sufficient to formally support surveillance discontinuation |

**Supplementary Table S2. Comparison of the 2017 Fukuoka and 2024 Kyoto IPMN frameworks and their implications for the present cohort**

This supplementary table summarizes the key differences between the 2017 revised Fukuoka guidelines and the 2024 Kyoto evidence-based guidelines for the management of intraductal papillary mucinous neoplasms (IPMNs). In addition, the table contextualizes these criteria within the present study cohort to facilitate interpretation of our findings in light of evolving international recommendations.

**Abbreviations:** BD-IPMN, branch-duct intraductal papillary mucinous neoplasm; HRS, high-risk stigmata; WF, worrisome features; MPD, main pancreatic duct.

When interpreted within the framework of the updated Kyoto 2024 guidelines, the outcomes observed in our cohort remain consistent with a predominantly indolent disease course in carefully selected low-risk BD-IPMN patients. The absence of HRS and the low rate of WF development support the concept of risk-adapted surveillance. However, the classification of cyst growth as a worrisome feature differs between frameworks, particularly with the introduction of an annualized growth threshold in the Kyoto guidelines. Importantly, while our data provide supportive evidence for potential surveillance de-escalation in small, stable cysts, the relatively limited median follow-up duration precludes definitive recommendations. These findings should therefore be interpreted as hypothesis-generating and require validation in larger, multicenter cohorts with longer follow-up.
